# Synthesis and crystal structures of 2-bromo-1,3-di­methyl­imidazolium iodides

**DOI:** 10.1107/S2056989018003390

**Published:** 2018-03-09

**Authors:** Martin Lampl, Gerhard Laus, Volker Kahlenberg, Klaus Wurst, Hubert Huppertz, Herwig Schottenberger

**Affiliations:** a University of Innsbruck, Faculty of Chemistry and Pharmacy, Innrain 80–82, 6020 Innsbruck, Austria; b University of Innsbruck, Institute of Mineralogy and Petrography, Innrain 52, 6020 Innsbruck, Austria

**Keywords:** crystal structure, bromo, chloro­form, di­chloro­methane, imidazole, iodide

## Abstract

Attempts at direct bromination of 1,3-di­methyl­imidazolium salts were futile. The title compounds, 2-bromo-1,3-di­methyl­imidazolium iodide chloro­form 0.33-solvate, C_5_H_8_BrN_2_^+^·I^−^·0.33CHCl_3_, 2-bromo-1,3-di­methyl­imidazolium iodide di­chloro­methane hemisolvate, C_5_H_8_BrN_2_^+^·I^−^·0.5CH_2_Cl_2_, and 2-bromo-1,3-di­methyl­imidazolium iodide hemi(diiodide), C_5_H_8_BrN_2_^+^·I^−^·0.5I_2_, were obtained by methyl­ation of 2-bromo-1-methyl­imidazole. They crystallized as CHCl_3_, CH_2_Cl_2_ or I_2_ solvates/adducts. The Br atom acts as a σ-hole to accept short C—Br⋯I inter­actions. C—H⋯I hydrogen bonds are observed in each structure.

## Abstract 2

Short C—Br⋯I inter­actions and C—H⋯I hydrogen bonds are observed in the title compounds.

## Chemical context   

Salts containing 2-bromo-1,3-di­methyl­imidazolium (C_5_H_8_N_2_Br^+^) cations are the objective of this work. They are presumed to be valuable precursors for substitution reactions. This cation, despite its simplicity, has not yet been described. Since brominations in the 1,3-di­meth­oxy­imidazolium series (Laus *et al.*, 2007 ▸) and also bromination of 1-hy­droxy­imidazole-3-oxide (Laus *et al.*, 2012 ▸) gave the respective 2-bromo derivatives, we hoped that in the present case bromination would also yield the desired 2-bromo­imidazolium salts. However, on attempted bromination of 1,3-di­methyl­imidazolium hexa­fluorido­phosphate (Holbrey *et al.*, 2002 ▸), no substitution occurred in the 2-position as indicated by NMR. The absence of P—F vibrations in the infrared spectra suggested the formation of a different anion, which was confirmed by X-ray diffraction. Though direct bromination of the quaternary salt did not yield the desired product, it was discovered that an altered sequence of reaction was successful. Thus, the reaction between the 2-li­thio derivative of 1-methyl­imidazole and an equimolar amount of CBr_4_ (Boga *et al.*, 2000 ▸) or Br_2_ (El Borai *et al.*, 1981 ▸) gave 2-bromo-1-methyl­imidazole in good yield, followed by methyl­ation using MeI to afford the desired quaternary salt as an iodide.

Now that the elusive title cation has been secured, further modifications are envisioned, giving access to a plethora of new 2-substituted imidazolium derivatives.

## Structural commentary   

**Figure 1:**
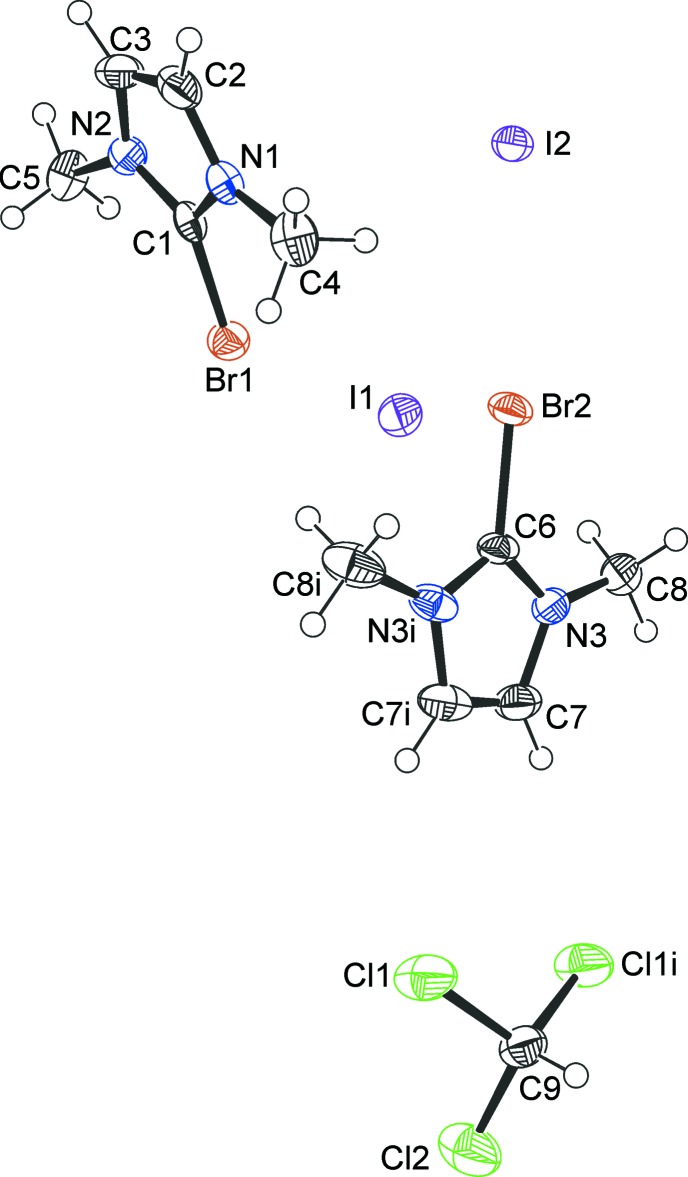
The mol­ecular structure of the chloro­form solvate **1**, showing the atom labels and 50% probability displacement ellipsoids for non-H atoms. [Symmetry code: (i) *x*, 1 − *y*, *z*.]

**Figure 2:**
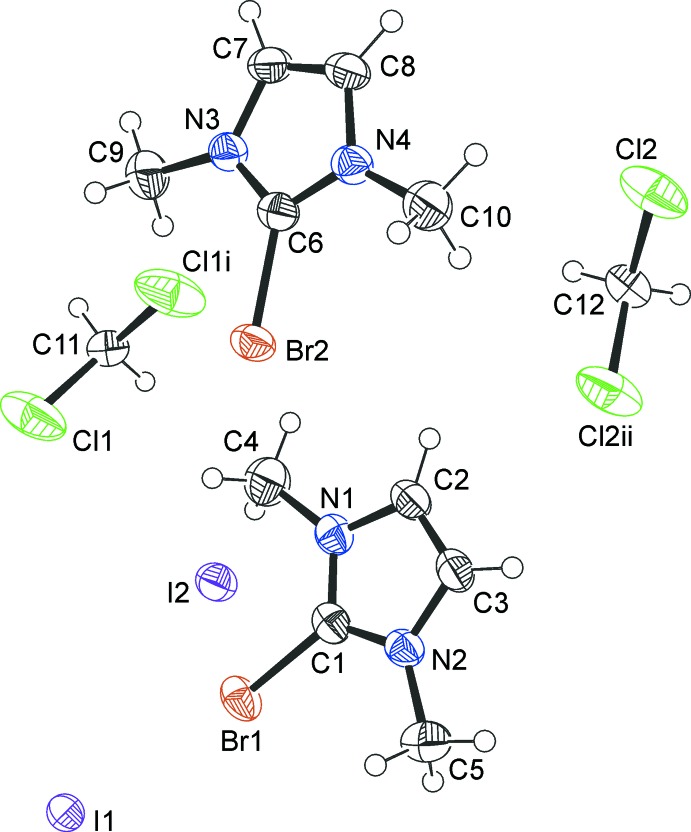
The mol­ecular structure of the iodide **2**, showing the atom labels and 50% probability displacement ellipsoids for non-H atoms. [Symmetry codes: (i) 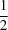 − *x*, *y*, 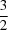 − *z*, (ii) 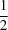 − *x*, *y*, 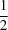 − *z*.]

**Figure 3:**
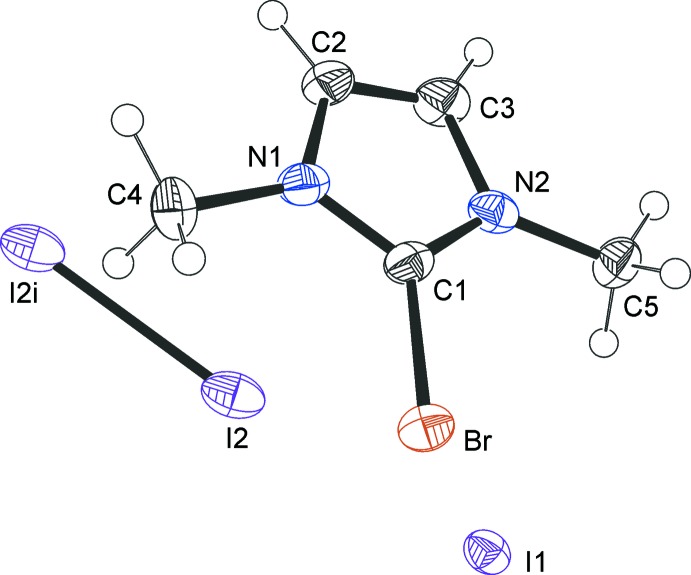
The mol­ecular structure of the iodide **3**, showing the atom labels and 50% probability displacement ellipsoids for non-H atoms. [Symmetry code: (i) 1 − *x*, 1 − *y*, −*z*.]

The 2-bromo-1,3-di­methyl­imidazolium cations and iodide counter-ions crystallize as a CHCl_3_ 1/3-solvate (**1**) (Fig. 1 ▸), a CH_2_Cl_2_ monosolvate (**2**) (Fig. 2 ▸) and an I_2_ adduct (**3**) (Fig. 3 ▸). In every case, the cation is almost planar. In the asymmetric unit of **1**, there are one and a half ion pairs, which are completed by mirror symmetry; the chloro­form mol­ecule also lies on a crystallographic mirror plane. In **2**, there are two cations, two anions and two half-mol­ecules of di­chloro­methane (both completed by crystallographic twofold symmetry) in the asymmetric unit. In **3**, the iodine mol­ecule is generated by crystallographic inversion symmetry.
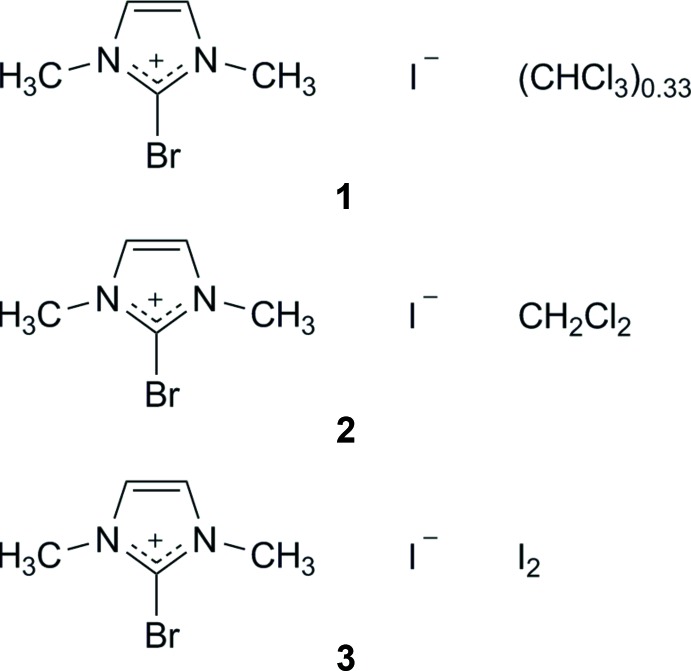


## Supra­molecular features   

**Figure 4:**
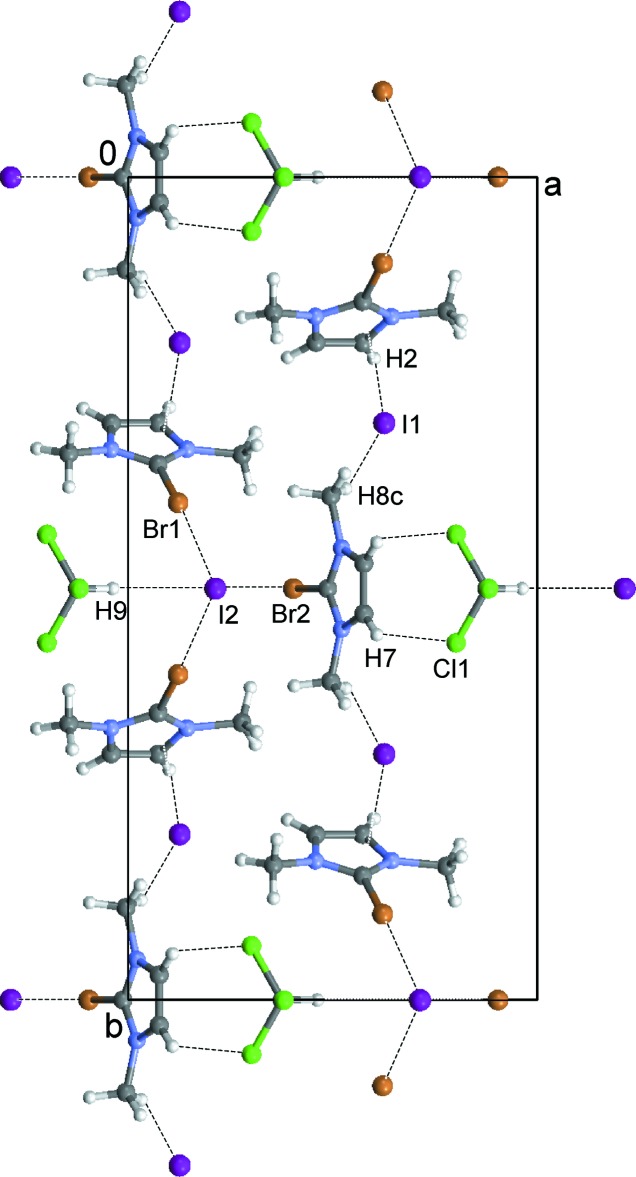
The crystal packing of compound **1** viewed along the *c* axis showing the C—H⋯Cl and C—H⋯I hydrogen bonds (see Table 1 ▸) and Br⋯I short contacts as dashed lines.

Halogen–halogen inter­actions constitute the main supra­molecular features of the three compounds. The cations in **1** are arranged in a tridimensional array of chains by C—H⋯I1 inter­actions. The chloro­form mol­ecule bridges these chains by C—H7⋯Cl1 and C—H9⋯I2 hydrogen bonds (Table 1 ▸). Inter­halogen Br1⋯I2(*x*, *y*, −1 + *z*) [3.544 (1) Å] and Br2⋯I2 [3.546 (2) Å] contacts complete the network (Fig. 4 ▸). The respective C—Br⋯I angles are 173.4 (2) and 173.6 (3)°, indicating an inter­action involving the positive end cap (σ-hole) of the terminal Br atom (Awwadi *et al.*, 2006 ▸; Clark *et al.*, 2007 ▸).

**Figure 5:**
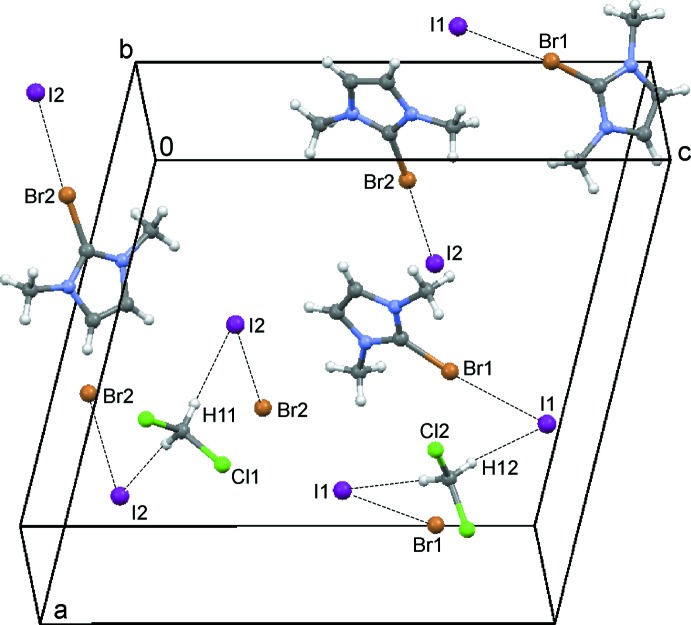
The crystal packing of compound **2** viewed along the *b* axis showing the C—H⋯I hydrogen bonds involving the solvent (see Table 2 ▸) and Br⋯I short contacts as dashed lines.

This type of inter­action is also identified in the structures of compounds **2** and **3**. In the di­chloro­methane solvate **2**, almost linear halogen inter­actions Br1⋯I1 [3.483 (1) Å] and Br2⋯I2 [3.411 (1) Å] exhibit C—Br⋯I angles of 173.7 (1) and 176.7 (1)°, respectively (Fig. 5 ▸). The I1 and I2 anions are linked by hydrogen bonds donated by the solvent mol­ecules (Table 2 ▸).

**Figure 6:**
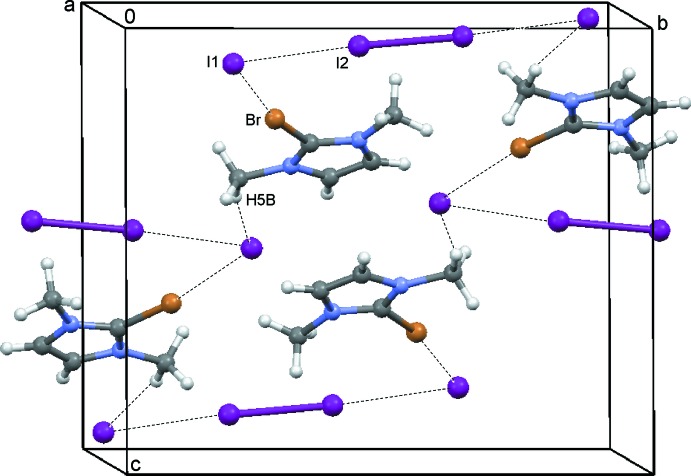
The crystal packing of compound **3** viewed along the *a* axis showing the C—H⋯I hydrogen bonds (see Table 3 ▸) and Br⋯I and I⋯I short contacts as dashed lines.

In **3**, a mol­ecular addition compound with iodine (Fig. 6 ▸), inter­actions I1⋯I2 [3.426 (1) Å] and I2—I2 [related by inversion, bond length 2.826 (1) Å] are present. The I1⋯Br(1 + *x*, *y*, *z*) [3.499 (1) Å] inter­action displays a C—Br⋯I angle of 168.0 (2)° (Fig. 6 ▸) and the iodide anion (I1) accepts a hydrogen bond from the methyl group (Table 3 ▸).

## Database survey   

A search of the Cambridge Structural Database (Version 5.38; Groom *et al.*, 2016 ▸) for 2-halogeno-1,3-dialkyl or di­aryl­imidazolium salts gave 30 hits. When carbon substituents were allowed in positions 4 and 5, the tally was 34. Of these 64 compounds, there were 11 containing chlorine, 19 bromine and 33 iodine. Closely related imidazolin-2-yl­idene–iodine (Kuhn *et al.*, 1993 ▸) and imidazolin-2-yl­idene–bromine (Kuhn *et al.*, 2004 ▸) coordination compounds have been reported.

## Synthesis and crystallization   

Compound **1**: A solution of 2-bromo-1-methyl­imidazole (150 µl, 1.54 mmol) in CHCl_3_ (1 ml) was carefully layered over a solution of CH_3_I (190 µl, 3.07 mmol) in CHCl_3_ (2 ml). The mixture was kept at room temperature and protected from light. After 2 h, the formation of colourless crystals of **1** was observed. The product was collected after seven days at 278 K, yielding 252 mg (48%); m.p. 453 K (decomposition). ^1^H NMR (300 MHz, DMSO-*d*_6_): δ 3.81 (*s*, 6H), 7.90 (*s*, 2H), 8.31 (*s*) ppm. ^13^C NMR (75 MHz, DMSO-*d*_6_): δ 36.8 (2C), 79.3, 123.5, 124.5 (2C) ppm. IR (neat): ν 3066, 2931, 1521, 1240, 1098, 765, 738, 652, 635 cm^−1^.

Compound **2**: A solution of 2-bromo-1-methyl­imidazole (150 µl, 1.54 mmol) in CH_2_Cl_2_ (1 ml) was carefully layered over a solution of CH_3_I (190 µl, 3.07 mmol) in CH_2_Cl_2_ (2 ml). The mixture was kept at room temperature and protected from light. After 2 h, the formation of colourless crystals of **2** was observed. The product was collected after 18 h, yielding 145 mg (27%); m.p. 452–453 K (decomposition). ^1^H NMR (300 MHz, DMSO-*d*_6_): δ 3.81 (*s*, 6H), 5.75, 7.90 (*s*, 2H) ppm. ^13^C NMR (75 MHz, DMSO-*d*_6_): δ 36.8 (2C), 55.0, 123.3, 124.7 (2C) ppm. IR (neat): ν 3066, 3011, 2944, 1523, 1240, 1101, 779, 728, 696, 635 cm^−1^.

Compound **3**: The I_2_ adduct was obtained as a byproduct of **1** and **2** in the form of brown crystals of **3**; approximate yield 10%; m.p. 451 K (decomposition). ^1^H NMR (300 MHz, DMSO-*d*_6_): δ 3.81 (*s*, 6H), 7.89 (*d*, 2H) ppm IR (neat): ν 3063, 1523, 1226, 739, 634 cm^−1^.

## Refinement   

Crystal data, data collection and structure refinement details are summarized in Table 4 ▸. All H atoms were poisitioned geometrically (C—H = 0.95–1.0 Å) and treated as riding with *U*_iso_(H) = 1.2–1.5*U*_eq_(C).

## Supplementary Material

Crystal structure: contains datablock(s) 1, 2, 3, global. DOI: 10.1107/S2056989018003390/hb7718sup1.cif

Structure factors: contains datablock(s) 1. DOI: 10.1107/S2056989018003390/hb77181sup2.hkl

Supporting information file. DOI: 10.1107/S2056989018003390/hb77181sup5.mol

Supporting information file. DOI: 10.1107/S2056989018003390/hb77181sup8.cml

Structure factors: contains datablock(s) 2. DOI: 10.1107/S2056989018003390/hb77182sup3.hkl

Supporting information file. DOI: 10.1107/S2056989018003390/hb77182sup6.mol

Supporting information file. DOI: 10.1107/S2056989018003390/hb77182sup9.cml

Supporting information file. DOI: 10.1107/S2056989018003390/hb77183sup10.cml

Structure factors: contains datablock(s) 3. DOI: 10.1107/S2056989018003390/hb77183sup4.hkl

Supporting information file. DOI: 10.1107/S2056989018003390/hb77183sup7.mol

CCDC references: 1826102, 1826101, 1826100

Additional supporting information:  crystallographic information; 3D view; checkCIF report

## Figures and Tables

**Table 1:** Hydrogen-bond geometry (Å, °) for **1** [Chem scheme1]

*D*—H⋯*A*	*D*—H	H⋯*A*	*D*⋯*A*	*D*—H⋯*A*
C7—H7⋯Cl1^i^	0.95	2.82	3.623 (7)	142
C8—H8*C*⋯I1^i^	0.98	3.02	3.935 (6)	156
C9—H9⋯I2^ii^	1.00	2.77	3.760 (8)	169
C2—H2⋯I1^iii^	0.95	3.01	3.932 (6)	165
C3—H3⋯I1^iv^	0.95	3.12	3.952 (9)	147

Symmetry codes: (i) 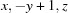; (ii) 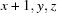; (iii) 
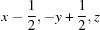
; (iv) 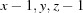.

**Table 2:** Hydrogen-bond geometry (Å, °) for **2** [Chem scheme1]

*D*—H⋯*A*	*D*—H	H⋯*A*	*D*⋯*A*	*D*—H⋯*A*
C11—H11*A*⋯I2^i^	0.99	2.86	3.834 (2)	169
C12—H12*A*⋯I1^ii^	0.99	2.89	3.862 (2)	170

Symmetry codes: (i) 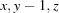; (ii) 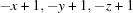.

**Table 3:** Hydrogen-bond geometry (Å, °) for **3** [Chem scheme1]

*D*—H⋯*A*	*D*—H	H⋯*A*	*D*⋯*A*	*D*—H⋯*A*
C5—H5*B*⋯I1^i^	0.98	3.03	3.986 (8)	166

Symmetry code: (i) 
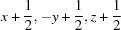
.

**Table 4:** Experimental details

	**1**	**2**	**3**
Crystal data			
Chemical formula	3C_5_H_8_BrN_2_^+^·3I^−^·CHCl_3_	2C_5_H_8_BrN_2_^+^·2I^−^·CH_2_Cl_2_	C_5_H_8_BrN_2_^+^·I^−^·0.5I_2_
*M*_r_	1028.20	690.79	429.83
Crystal system, space group	Monoclinic, *C* *m*	Monoclinic, *P*2/*n*	Monoclinic, *P*2_1_/*n*
Temperature (K)	173	193	173
*a*, *b*, *c* (Å)	13.9135 (14), 21.9492 (10), 6.4529 (6)	16.0223 (8), 8.5334 (4), 16.2881 (8)	6.0861 (4), 14.4773 (11), 12.0303 (7)
β (°)	128.314 (16)	101.590 (1)	97.812 (5)
*V* (Å^3^)	1546.2 (3)	2181.58 (18)	1050.16 (12)
*Z*	2	4	4
Radiation type	Mo *K*α	Mo *K*α	Mo *K*α
μ (mm^−1^)	7.18	6.79	9.74
Crystal size (mm)	0.26 × 0.14 × 0.06	0.18 × 0.16 × 0.14	0.36 × 0.10 × 0.08

Data collection			
Diffractometer	Gemini-R Ultra	Quest Photon 100	Gemini-R Ultra
Absorption correction	Multi-scan (*CrysAlis PRO*; Oxford Diffraction, 2014 ▸)	Multi-scan (*SADABS*; Bruker, 2014 ▸)	Analytical
*T*_min_, *T*_max_	0.427, 1	0.296, 0.433	0.065, 0.446
No. of measured, independent and observed [*I* > 2σ(*I*)] reflections	4904, 2522, 2426	62055, 4302, 3952	6287, 1912, 1746
*R*_int_	0.026	0.028	0.030
(sin θ/λ)_max_ (Å^−1^)	0.602	0.617	0.602

Refinement			
*R*[*F* ^2^ > 2σ(*F* ^2^)], *wR*(*F* ^2^), *S*	0.018, 0.035, 0.95	0.022, 0.063, 1.09	0.036, 0.080, 1.34
No. of reflections	2522	4302	1912
No. of parameters	151	196	93
No. of restraints	2	0	0
H-atom treatment	H-atom parameters constrained	H-atom parameters constrained	H-atom parameters constrained
Δρ_max_, Δρ_min_ (e Å^−3^)	0.41, −0.44	1.07, −0.76	0.76, −0.97
Absolute structure	Flack *x* determined using 961 quotients [(*I* ^+^)−(*I* ^−^)]/[(*I* ^+^)+(*I* ^−^)] (Parsons *et al.*, 2013 ▸)	–	–
Absolute structure parameter	0.038 (8)	–	–

Computer programs: *APEX2* and *SAINT* (Bruker, 2014 ▸), *CrysAlis PRO* (Oxford Diffraction, 2014 ▸), *SIR2002* (Burla *et al.*, 2003 ▸), *SHELXTL* (Sheldrick, 2008 ▸), *SHELXL2014* and *SHELXL2017* (Sheldrick, 2015 ▸), *ORTEP-3 for Windows* (Farrugia, 2012 ▸) and *Mercury* (Macrae *et al.*, 2008 ▸).

## supplementary crystallographic information

### 2-Bromo-1,3-dimethylimidazolium iodide chloroform 0.33-solvate (1) Crystal data

**Table:**

3C_5_H_8_BrN_2_^+^·3I^−^·CHCl_3_	*F*(000) = 956
*M**_r_* = 1028.20	*D*_x_ = 2.208 Mg m^−^^3^
Monoclinic, *C**m*	Mo *K*α radiation, λ = 0.71073 Å
*a* = 13.9135 (14) Å	Cell parameters from 3263 reflections
*b* = 21.9492 (10) Å	θ = 3.3–28.4°
*c* = 6.4529 (6) Å	µ = 7.18 mm^−^^1^
β = 128.314 (16)°	*T* = 173 K
*V* = 1546.2 (3) Å^3^	Prismatic, colourless
*Z* = 2	0.26 × 0.14 × 0.06 mm

### 2-Bromo-1,3-dimethylimidazolium iodide chloroform 0.33-solvate (1) Data collection

**Table:**

Gemini-R Ultra diffractometer	2426 reflections with *I* > 2σ(*I*)
Radiation source: Enhance (Mo) X-ray Source	*R*_int_ = 0.026
ω scans	θ_max_ = 25.3°, θ_min_ = 3.4°
Absorption correction: multi-scan (CrysAlis PRO; Oxford Diffraction, 2014)	*h* = −16→13
*T*_min_ = 0.427, *T*_max_ = 1	*k* = −22→26
4904 measured reflections	*l* = −7→7
2522 independent reflections	

### 2-Bromo-1,3-dimethylimidazolium iodide chloroform 0.33-solvate (1) Refinement

**Table:**

Refinement on *F*^2^	Hydrogen site location: inferred from neighbouring sites
Least-squares matrix: full	H-atom parameters constrained
*R*[*F*^2^ > 2σ(*F*^2^)] = 0.018	*w* = 1/[σ^2^(*F*_o_^2^) + (0.0049*P*)^2^] where *P* = (*F*_o_^2^ + 2*F*_c_^2^)/3
*wR*(*F*^2^) = 0.035	(Δ/σ)_max_ < 0.001
*S* = 0.95	Δρ_max_ = 0.41 e Å^−^^3^
2522 reflections	Δρ_min_ = −0.43 e Å^−^^3^
151 parameters	Absolute structure: Flack *x* determined using 961 quotients [(*I*^+^)-(*I*^-^)]/[(*I*^+^)+(*I*^-^)] (Parsons *et al.*, 2013)
2 restraints	Absolute structure parameter: 0.038 (8)

### 2-Bromo-1,3-dimethylimidazolium iodide chloroform 0.33-solvate (1) Special details

**Table:**

Geometry. All esds (except the esd in the dihedral angle between two l.s. planes) are estimated using the full covariance matrix. The cell esds are taken into account individually in the estimation of esds in distances, angles and torsion angles; correlations between esds in cell parameters are only used when they are defined by crystal symmetry. An approximate (isotropic) treatment of cell esds is used for estimating esds involving l.s. planes.

### 2-Bromo-1,3-dimethylimidazolium iodide chloroform 0.33-solvate (1) Fractional atomic coordinates and isotropic or equivalent isotropic displacement parameters (Å^2^)

**Table:**

	*x*	*y*	*z*	*U*_iso_*/*U*_eq_	
Br1	0.12194 (6)	0.39565 (3)	−0.07927 (14)	0.03269 (15)	
C2	0.0731 (6)	0.2987 (3)	0.3714 (13)	0.0354 (15)	
H2	0.100922	0.279634	0.532098	0.042*	
C3	−0.0414 (6)	0.2992 (3)	0.1483 (14)	0.0353 (16)	
H3	−0.110268	0.280970	0.121251	0.042*	
C1	0.0731 (5)	0.3488 (2)	0.0790 (11)	0.0246 (14)	
N1	0.1435 (4)	0.3305 (2)	0.3284 (9)	0.0284 (11)	
C4	0.2778 (6)	0.3372 (3)	0.5169 (14)	0.0405 (17)	
H4A	0.313345	0.325857	0.430407	0.061*	
H4B	0.310751	0.310582	0.669397	0.061*	
H4C	0.298588	0.379637	0.576266	0.061*	
N2	−0.0420 (4)	0.3308 (2)	−0.0364 (10)	0.0283 (12)	
C5	−0.1452 (5)	0.3358 (3)	−0.3220 (12)	0.0353 (15)	
H5A	−0.144701	0.376365	−0.385102	0.053*	
H5B	−0.222661	0.329753	−0.351574	0.053*	
H5C	−0.136654	0.304732	−0.418510	0.053*	
I1	0.62590 (4)	0.29885 (2)	0.94514 (6)	0.02823 (10)	
I2	0.21304 (5)	0.500000	0.65285 (9)	0.02890 (13)	
Br2	0.40367 (7)	0.500000	0.46275 (15)	0.0313 (2)	
C8	0.4948 (7)	0.6124 (3)	0.2888 (14)	0.0462 (18)	
H8A	0.405961	0.618288	0.177487	0.069*	
H8B	0.529339	0.639748	0.230124	0.069*	
H8C	0.531481	0.621371	0.472955	0.069*	
N3	0.5211 (4)	0.5494 (2)	0.2675 (9)	0.0315 (12)	
C6	0.4874 (7)	0.500000	0.3242 (15)	0.0269 (19)	
C7	0.5758 (6)	0.5305 (3)	0.1602 (12)	0.0399 (16)	
H7	0.607822	0.556086	0.097268	0.048*	
C9	0.8764 (8)	0.500000	0.0794 (19)	0.038 (2)	
H9	0.962304	0.500000	0.247965	0.045*	
Cl1	0.8016 (2)	0.43407 (7)	0.0686 (5)	0.0569 (5)	
Cl2	0.8806 (3)	0.500000	−0.1852 (6)	0.0636 (8)	

### 2-Bromo-1,3-dimethylimidazolium iodide chloroform 0.33-solvate (1) Atomic displacement parameters (Å^2^)

**Table:**

	*U*^11^	*U*^22^	*U*^33^	*U*^12^	*U*^13^	*U*^23^
Br1	0.0383 (3)	0.0293 (3)	0.0368 (3)	−0.0004 (3)	0.0265 (3)	0.0013 (3)
C2	0.052 (4)	0.032 (3)	0.033 (4)	0.001 (3)	0.031 (4)	−0.001 (3)
C3	0.046 (4)	0.031 (4)	0.050 (4)	0.002 (3)	0.040 (4)	0.003 (3)
C1	0.029 (3)	0.019 (3)	0.023 (3)	0.003 (3)	0.015 (3)	−0.003 (3)
N1	0.032 (3)	0.025 (3)	0.026 (3)	0.003 (2)	0.017 (2)	−0.003 (2)
C4	0.043 (4)	0.032 (3)	0.038 (4)	0.006 (3)	0.021 (4)	0.002 (3)
N2	0.028 (3)	0.024 (3)	0.035 (3)	0.002 (2)	0.021 (2)	−0.001 (2)
C5	0.022 (3)	0.033 (3)	0.031 (4)	0.003 (3)	0.007 (3)	0.005 (3)
I1	0.03254 (19)	0.02663 (18)	0.0289 (2)	−0.00359 (18)	0.02071 (17)	0.00012 (18)
I2	0.0286 (3)	0.0344 (3)	0.0280 (3)	0.000	0.0196 (3)	0.000
Br2	0.0373 (5)	0.0385 (5)	0.0292 (5)	0.000	0.0262 (4)	0.000
C8	0.059 (5)	0.044 (4)	0.052 (5)	−0.021 (3)	0.043 (4)	−0.015 (3)
N3	0.028 (3)	0.045 (3)	0.023 (3)	−0.011 (2)	0.016 (2)	−0.005 (2)
C6	0.021 (4)	0.044 (5)	0.018 (4)	0.000	0.013 (4)	0.000
C7	0.031 (3)	0.064 (4)	0.031 (4)	−0.008 (3)	0.022 (3)	−0.004 (3)
C9	0.034 (5)	0.031 (5)	0.053 (6)	0.000	0.030 (5)	0.000
Cl1	0.0748 (12)	0.0285 (7)	0.1059 (15)	0.0015 (10)	0.0751 (12)	0.0040 (11)
Cl2	0.102 (2)	0.0363 (14)	0.097 (2)	0.000	0.084 (2)	0.000

### 2-Bromo-1,3-dimethylimidazolium iodide chloroform 0.33-solvate (1) Geometric parameters (Å, º)

**Table:**

Br1—C1	1.850 (6)	C5—H5C	0.9800
C2—C3	1.329 (9)	Br2—C6	1.857 (8)
C2—N1	1.364 (8)	C8—N3	1.457 (8)
C2—H2	0.9500	C8—H8A	0.9800
C3—N2	1.374 (8)	C8—H8B	0.9800
C3—H3	0.9500	C8—H8C	0.9800
C1—N1	1.325 (7)	N3—C6	1.321 (6)
C1—N2	1.340 (7)	N3—C7	1.372 (7)
N1—C4	1.475 (8)	C7—C7^i^	1.340 (13)
C4—H4A	0.9800	C7—H7	0.9500
C4—H4B	0.9800	C9—Cl2	1.744 (9)
C4—H4C	0.9800	C9—Cl1^i^	1.759 (5)
N2—C5	1.480 (8)	C9—Cl1	1.759 (5)
C5—H5A	0.9800	C9—H9	1.0000
C5—H5B	0.9800		
			
C3—C2—N1	107.7 (6)	N2—C5—H5C	109.5
C3—C2—H2	126.1	H5A—C5—H5C	109.5
N1—C2—H2	126.1	H5B—C5—H5C	109.5
C2—C3—N2	107.6 (6)	N3—C8—H8A	109.5
C2—C3—H3	126.2	N3—C8—H8B	109.5
N2—C3—H3	126.2	H8A—C8—H8B	109.5
N1—C1—N2	108.3 (5)	N3—C8—H8C	109.5
N1—C1—Br1	126.2 (4)	H8A—C8—H8C	109.5
N2—C1—Br1	125.3 (4)	H8B—C8—H8C	109.5
C1—N1—C2	108.6 (5)	C6—N3—C7	107.1 (5)
C1—N1—C4	125.2 (5)	C6—N3—C8	126.8 (5)
C2—N1—C4	125.8 (5)	C7—N3—C8	125.8 (5)
N1—C4—H4A	109.5	N3^i^—C6—N3	110.5 (7)
N1—C4—H4B	109.5	N3^i^—C6—Br2	124.8 (4)
H4A—C4—H4B	109.5	N3—C6—Br2	124.8 (4)
N1—C4—H4C	109.5	C7^i^—C7—N3	107.6 (4)
H4A—C4—H4C	109.5	C7^i^—C7—H7	126.2
H4B—C4—H4C	109.5	N3—C7—H7	126.2
C1—N2—C3	107.7 (5)	Cl2—C9—Cl1^i^	109.8 (4)
C1—N2—C5	125.0 (5)	Cl2—C9—Cl1	109.8 (4)
C3—N2—C5	126.6 (5)	Cl1^i^—C9—Cl1	110.8 (5)
N2—C5—H5A	109.5	Cl2—C9—H9	108.8
N2—C5—H5B	109.5	Cl1^i^—C9—H9	108.8
H5A—C5—H5B	109.5	Cl1—C9—H9	108.8
			
N1—C2—C3—N2	0.8 (7)	Br1—C1—N2—C5	11.5 (8)
N2—C1—N1—C2	1.8 (6)	C2—C3—N2—C1	0.3 (7)
Br1—C1—N1—C2	178.1 (4)	C2—C3—N2—C5	171.0 (5)
N2—C1—N1—C4	175.1 (5)	C7—N3—C6—N3^i^	2.1 (8)
Br1—C1—N1—C4	−8.6 (8)	C8—N3—C6—N3^i^	176.4 (4)
C3—C2—N1—C1	−1.6 (7)	C7—N3—C6—Br2	−177.6 (5)
C3—C2—N1—C4	−174.9 (6)	C8—N3—C6—Br2	−3.3 (10)
N1—C1—N2—C3	−1.3 (6)	C6—N3—C7—C7^i^	−1.3 (5)
Br1—C1—N2—C3	−177.6 (4)	C8—N3—C7—C7^i^	−175.7 (5)
N1—C1—N2—C5	−172.2 (5)		

Symmetry code: (i) *x*, −*y*+1, *z*.

### 2-Bromo-1,3-dimethylimidazolium iodide chloroform 0.33-solvate (1) Hydrogen-bond geometry (Å, º)

**Table:**

*D*—H···*A*	*D*—H	H···*A*	*D*···*A*	*D*—H···*A*
C7—H7···Cl1^i^	0.95	2.82	3.623 (7)	142
C8—H8*C*···I1^i^	0.98	3.02	3.935 (6)	156
C9—H9···I2^ii^	1.00	2.77	3.760 (8)	169
C2—H2···I1^iii^	0.95	3.01	3.932 (6)	165
C3—H3···I1^iv^	0.95	3.12	3.952 (9)	147

Symmetry codes: (i) *x*, −*y*+1, *z*; (ii) *x*+1, *y*, *z*; (iii) *x*−1/2, −*y*+1/2, *z*; (iv) *x*−1, *y*, *z*−1.

### 2-Bromo-1,3-dimethylimidazolium iodide dichloromethane hemisolvate (2) Crystal data

**Table:**

2C_5_H_8_BrN_2_^+^·2I^−^·CH_2_Cl_2_	*F*(000) = 1288
*M**_r_* = 690.79	*D*_x_ = 2.103 Mg m^−^^3^
Monoclinic, *P*2/*n*	Mo *K*α radiation, λ = 0.71073 Å
*a* = 16.0223 (8) Å	Cell parameters from 9539 reflections
*b* = 8.5334 (4) Å	θ = 2.5–26.8°
*c* = 16.2881 (8) Å	µ = 6.79 mm^−^^1^
β = 101.590 (1)°	*T* = 193 K
*V* = 2181.58 (18) Å^3^	Prism, colourless
*Z* = 4	0.18 × 0.16 × 0.14 mm

### 2-Bromo-1,3-dimethylimidazolium iodide dichloromethane hemisolvate (2) Data collection

**Table:**

Quest Photon 100 diffractometer	4302 independent reflections
Radiation source: Incoatec Microfocus	3952 reflections with *I* > 2σ(*I*)
Multi layered optics monochromator	*R*_int_ = 0.028
Detector resolution: 10.4 pixels mm^-1^	θ_max_ = 26.0°, θ_min_ = 2.4°
φ and ω scans	*h* = −19→19
Absorption correction: multi-scan (SADABS; Bruker, 2014)	*k* = −10→10
*T*_min_ = 0.296, *T*_max_ = 0.433	*l* = −20→20
62055 measured reflections	

### 2-Bromo-1,3-dimethylimidazolium iodide dichloromethane hemisolvate (2) Refinement

**Table:**

Refinement on *F*^2^	Hydrogen site location: inferred from neighbouring sites
Least-squares matrix: full	H-atom parameters constrained
*R*[*F*^2^ > 2σ(*F*^2^)] = 0.022	*w* = 1/[σ^2^(*F*_o_^2^) + (0.0332*P*)^2^ + 2.6592*P*] where *P* = (*F*_o_^2^ + 2*F*_c_^2^)/3
*wR*(*F*^2^) = 0.063	(Δ/σ)_max_ = 0.001
*S* = 1.09	Δρ_max_ = 1.07 e Å^−^^3^
4302 reflections	Δρ_min_ = −0.76 e Å^−^^3^
196 parameters	Extinction correction: SHELXL2014 (Sheldrick, 2015), Fc^*^=kFc[1+0.001xFc^2^λ^3^/sin(2θ)]^-1/4^
0 restraints	Extinction coefficient: 0.00234 (11)

### 2-Bromo-1,3-dimethylimidazolium iodide dichloromethane hemisolvate (2) Special details

**Table:**

Geometry. All esds (except the esd in the dihedral angle between two l.s. planes) are estimated using the full covariance matrix. The cell esds are taken into account individually in the estimation of esds in distances, angles and torsion angles; correlations between esds in cell parameters are only used when they are defined by crystal symmetry. An approximate (isotropic) treatment of cell esds is used for estimating esds involving l.s. planes.

### 2-Bromo-1,3-dimethylimidazolium iodide dichloromethane hemisolvate (2) Fractional atomic coordinates and isotropic or equivalent isotropic displacement parameters (Å^2^)

**Table:**

	*x*	*y*	*z*	*U*_iso_*/*U*_eq_	Occ. (<1)
I1	0.67871 (2)	0.51565 (2)	0.93259 (2)	0.03524 (8)	
I2	0.43487 (2)	1.00139 (2)	0.67926 (2)	0.03507 (8)	
Br1	0.58435 (2)	0.60778 (4)	0.72654 (2)	0.04235 (10)	
Br2	0.23431 (2)	0.90371 (4)	0.57720 (2)	0.03775 (10)	
N1	0.44318 (17)	0.5987 (3)	0.58815 (16)	0.0365 (6)	
N2	0.54830 (15)	0.7333 (3)	0.55936 (15)	0.0345 (5)	
N3	0.06441 (15)	0.7732 (3)	0.54179 (15)	0.0344 (5)	
N4	0.09163 (16)	0.9098 (3)	0.43839 (16)	0.0347 (5)	
C1	0.52208 (19)	0.6502 (4)	0.61831 (18)	0.0347 (6)	
C2	0.4178 (2)	0.6538 (4)	0.50716 (19)	0.0387 (7)	
H2	0.3644	0.6356	0.4708	0.046*	
C3	0.4831 (2)	0.7381 (4)	0.48965 (19)	0.0389 (7)	
H3	0.4841	0.7913	0.4386	0.047*	
C4	0.3892 (3)	0.5077 (5)	0.6337 (3)	0.0550 (10)	
H4A	0.4211	0.4167	0.6602	0.083*	
H4B	0.3382	0.4720	0.5945	0.083*	
H4C	0.3724	0.5737	0.6769	0.083*	
C5	0.6292 (2)	0.8180 (4)	0.5680 (2)	0.0494 (8)	
H5A	0.6345	0.8927	0.6145	0.074*	
H5B	0.6305	0.8747	0.5160	0.074*	
H5C	0.6765	0.7433	0.5793	0.074*	
C6	0.12335 (18)	0.8585 (3)	0.51581 (19)	0.0334 (6)	
C7	−0.00661 (19)	0.7668 (4)	0.4784 (2)	0.0383 (7)	
H7	−0.0580	0.7122	0.4797	0.046*	
C8	0.01002 (19)	0.8519 (4)	0.4140 (2)	0.0387 (7)	
H8	−0.0274	0.8689	0.3617	0.046*	
C9	0.0746 (2)	0.6917 (5)	0.6219 (2)	0.0503 (8)	
H9A	0.1254	0.6251	0.6298	0.075*	
H9B	0.0243	0.6266	0.6224	0.075*	
H9C	0.0809	0.7686	0.6674	0.075*	
C10	0.1357 (3)	1.0097 (5)	0.3880 (3)	0.0530 (10)	
H10A	0.1510	1.1091	0.4172	0.080*	
H10B	0.0982	1.0301	0.3336	0.080*	
H10C	0.1875	0.9569	0.3792	0.080*	
C11	0.2500	0.2120 (5)	0.7500	0.0409 (10)	
H11A	0.2931	0.1437	0.7324	0.049*	0.5
H11B	0.2069	0.1436	0.7676	0.049*	0.5
Cl1	0.29912 (8)	0.32404 (14)	0.83510 (8)	0.0829 (4)	
C12	0.2500	0.7005 (5)	0.2500	0.0416 (10)	
H12A	0.2689	0.6322	0.2081	0.050*	0.5
H12B	0.2311	0.6322	0.2919	0.050*	0.5
Cl2	0.16400 (7)	0.81502 (12)	0.20000 (7)	0.0695 (3)	

### 2-Bromo-1,3-dimethylimidazolium iodide dichloromethane hemisolvate (2) Atomic displacement parameters (Å^2^)

**Table:**

	*U*^11^	*U*^22^	*U*^33^	*U*^12^	*U*^13^	*U*^23^
I1	0.03410 (12)	0.03872 (13)	0.03155 (12)	0.00227 (8)	0.00338 (9)	0.00373 (7)
I2	0.03022 (12)	0.04024 (13)	0.03290 (12)	0.00221 (7)	0.00191 (8)	−0.00410 (7)
Br1	0.04481 (19)	0.04589 (19)	0.03212 (17)	0.01593 (14)	−0.00236 (13)	0.00018 (13)
Br2	0.03094 (16)	0.04058 (18)	0.03833 (17)	−0.00138 (12)	−0.00111 (12)	−0.01062 (12)
N1	0.0399 (14)	0.0358 (14)	0.0331 (13)	0.0018 (11)	0.0057 (11)	−0.0024 (10)
N2	0.0315 (12)	0.0374 (13)	0.0331 (13)	0.0056 (10)	0.0025 (10)	−0.0051 (11)
N3	0.0320 (13)	0.0379 (13)	0.0326 (13)	0.0021 (10)	0.0050 (10)	−0.0042 (11)
N4	0.0326 (13)	0.0356 (13)	0.0349 (13)	0.0003 (10)	0.0046 (10)	−0.0052 (10)
C1	0.0382 (16)	0.0325 (15)	0.0317 (15)	0.0085 (12)	0.0032 (12)	−0.0038 (12)
C2	0.0384 (16)	0.0429 (17)	0.0315 (15)	0.0033 (14)	−0.0004 (12)	−0.0052 (13)
C3	0.0413 (17)	0.0454 (18)	0.0282 (15)	0.0076 (14)	0.0026 (12)	−0.0018 (13)
C4	0.058 (2)	0.057 (2)	0.051 (2)	−0.0060 (17)	0.0125 (19)	0.0077 (17)
C5	0.0364 (17)	0.054 (2)	0.055 (2)	−0.0011 (15)	0.0048 (15)	−0.0014 (17)
C6	0.0317 (14)	0.0315 (14)	0.0356 (15)	0.0037 (12)	0.0032 (12)	−0.0082 (12)
C7	0.0270 (14)	0.0438 (18)	0.0428 (17)	−0.0011 (12)	0.0039 (12)	−0.0061 (14)
C8	0.0306 (15)	0.0435 (17)	0.0383 (16)	0.0011 (13)	−0.0019 (12)	−0.0042 (14)
C9	0.054 (2)	0.058 (2)	0.0390 (18)	−0.0011 (17)	0.0089 (16)	0.0067 (16)
C10	0.051 (2)	0.061 (2)	0.046 (2)	−0.0109 (17)	0.0077 (17)	0.0060 (16)
C11	0.034 (2)	0.042 (2)	0.046 (3)	0.000	0.0059 (19)	0.000
Cl1	0.0724 (7)	0.0690 (7)	0.0889 (8)	0.0138 (6)	−0.0277 (6)	−0.0346 (6)
C12	0.044 (2)	0.040 (2)	0.037 (2)	0.000	−0.0023 (19)	0.000
Cl2	0.0684 (6)	0.0583 (6)	0.0657 (6)	0.0183 (5)	−0.0249 (5)	−0.0070 (5)

### 2-Bromo-1,3-dimethylimidazolium iodide dichloromethane hemisolvate (2) Geometric parameters (Å, º)

**Table:**

Br1—C1	1.878 (3)	C4—H4C	0.9800
Br2—C6	1.896 (3)	C5—H5A	0.9800
N1—C1	1.335 (4)	C5—H5B	0.9800
N1—C2	1.382 (4)	C5—H5C	0.9800
N1—C4	1.469 (5)	C7—C8	1.345 (5)
N2—C1	1.328 (4)	C7—H7	0.9500
N2—C3	1.380 (4)	C8—H8	0.9500
N2—C5	1.465 (4)	C9—H9A	0.9800
N3—C6	1.327 (4)	C9—H9B	0.9800
N3—C7	1.376 (4)	C9—H9C	0.9800
N3—C9	1.459 (4)	C10—H10A	0.9800
N4—C6	1.335 (4)	C10—H10B	0.9800
N4—C8	1.380 (4)	C10—H10C	0.9800
N4—C10	1.460 (4)	C11—Cl1	1.737 (3)
C2—C3	1.347 (5)	C11—H11A	0.9900
C2—H2	0.9500	C11—H11B	0.9900
C3—H3	0.9500	C12—Cl2	1.751 (3)
C4—H4A	0.9800	C12—H12A	0.9900
C4—H4B	0.9800	C12—H12B	0.9900
			
C1—N1—C2	108.3 (3)	N3—C6—N4	108.7 (3)
C1—N1—C4	126.7 (3)	N3—C6—Br2	126.5 (2)
C2—N1—C4	124.8 (3)	N4—C6—Br2	124.7 (2)
C1—N2—C3	108.3 (3)	C8—C7—N3	107.5 (3)
C1—N2—C5	126.6 (3)	C8—C7—H7	126.3
C3—N2—C5	124.9 (3)	N3—C7—H7	126.3
C6—N3—C7	108.5 (3)	C7—C8—N4	107.1 (3)
C6—N3—C9	126.0 (3)	C7—C8—H8	126.4
C7—N3—C9	125.4 (3)	N4—C8—H8	126.4
C6—N4—C8	108.2 (3)	N3—C9—H9A	109.5
C6—N4—C10	126.0 (3)	N3—C9—H9B	109.5
C8—N4—C10	125.8 (3)	H9A—C9—H9B	109.5
N2—C1—N1	108.9 (3)	N3—C9—H9C	109.5
N2—C1—Br1	126.6 (2)	H9A—C9—H9C	109.5
N1—C1—Br1	124.5 (2)	H9B—C9—H9C	109.5
C3—C2—N1	106.9 (3)	N4—C10—H10A	109.5
C3—C2—H2	126.5	N4—C10—H10B	109.5
N1—C2—H2	126.5	H10A—C10—H10B	109.5
C2—C3—N2	107.6 (3)	N4—C10—H10C	109.5
C2—C3—H3	126.2	H10A—C10—H10C	109.5
N2—C3—H3	126.2	H10B—C10—H10C	109.5
N1—C4—H4A	109.5	Cl1^i^—C11—Cl1	113.2 (3)
N1—C4—H4B	109.5	Cl1^i^—C11—H11A	108.9
H4A—C4—H4B	109.5	Cl1—C11—H11A	108.9
N1—C4—H4C	109.5	Cl1^i^—C11—H11B	108.9
H4A—C4—H4C	109.5	Cl1—C11—H11B	108.9
H4B—C4—H4C	109.5	H11A—C11—H11B	107.7
N2—C5—H5A	109.5	Cl2—C12—Cl2^ii^	112.1 (3)
N2—C5—H5B	109.5	Cl2—C12—H12A	109.2
H5A—C5—H5B	109.5	Cl2^ii^—C12—H12A	109.2
N2—C5—H5C	109.5	Cl2—C12—H12B	109.2
H5A—C5—H5C	109.5	Cl2^ii^—C12—H12B	109.2
H5B—C5—H5C	109.5	H12A—C12—H12B	107.9
			
C3—N2—C1—N1	1.4 (3)	C7—N3—C6—N4	1.4 (3)
C5—N2—C1—N1	176.4 (3)	C9—N3—C6—N4	177.9 (3)
C3—N2—C1—Br1	−179.2 (2)	C7—N3—C6—Br2	−179.2 (2)
C5—N2—C1—Br1	−4.3 (4)	C9—N3—C6—Br2	−2.6 (4)
C2—N1—C1—N2	−1.1 (3)	C8—N4—C6—N3	−1.2 (3)
C4—N1—C1—N2	−177.4 (3)	C10—N4—C6—N3	178.9 (3)
C2—N1—C1—Br1	179.5 (2)	C8—N4—C6—Br2	179.3 (2)
C4—N1—C1—Br1	3.2 (4)	C10—N4—C6—Br2	−0.5 (4)
C1—N1—C2—C3	0.3 (3)	C6—N3—C7—C8	−1.0 (3)
C4—N1—C2—C3	176.7 (3)	C9—N3—C7—C8	−177.5 (3)
N1—C2—C3—N2	0.5 (3)	N3—C7—C8—N4	0.2 (4)
C1—N2—C3—C2	−1.2 (3)	C6—N4—C8—C7	0.6 (3)
C5—N2—C3—C2	−176.2 (3)	C10—N4—C8—C7	−179.6 (3)

Symmetry codes: (i) −*x*+1/2, *y*, −*z*+3/2; (ii) −*x*+1/2, *y*, −*z*+1/2.

### 2-Bromo-1,3-dimethylimidazolium iodide dichloromethane hemisolvate (2) Hydrogen-bond geometry (Å, º)

**Table:**

*D*—H···*A*	*D*—H	H···*A*	*D*···*A*	*D*—H···*A*
C11—H11*A*···I2^iii^	0.99	2.86	3.834 (2)	169
C12—H12*A*···I1^iv^	0.99	2.89	3.862 (2)	170

Symmetry codes: (iii) *x*, *y*−1, *z*; (iv) −*x*+1, −*y*+1, −*z*+1.

### 2-Bromo-1,3-dimethylimidazolium iodide hemi(diiodide) (3) Crystal data

**Table:**

C_5_H_8_BrN_2_^+^·I^−^·0.5I_2_	*F*(000) = 772
*M**_r_* = 429.83	*D*_x_ = 2.719 Mg m^−^^3^
Monoclinic, *P*2_1_/*n*	Mo *K*α radiation, λ = 0.71073 Å
*a* = 6.0861 (4) Å	Cell parameters from 3142 reflections
*b* = 14.4773 (11) Å	θ = 3.3–27.6°
*c* = 12.0303 (7) Å	µ = 9.74 mm^−^^1^
β = 97.812 (5)°	*T* = 173 K
*V* = 1050.16 (12) Å^3^	Lath shaped, red-brown
*Z* = 4	0.36 × 0.10 × 0.08 mm

### 2-Bromo-1,3-dimethylimidazolium iodide hemi(diiodide) (3) Data collection

**Table:**

Gemini-R Ultra diffractometer	1746 reflections with *I* > 2σ(*I*)
ω scans	*R*_int_ = 0.030
Absorption correction: analytical	θ_max_ = 25.4°, θ_min_ = 3.3°
*T*_min_ = 0.065, *T*_max_ = 0.446	*h* = −6→7
6287 measured reflections	*k* = −17→13
1912 independent reflections	*l* = −14→11

### 2-Bromo-1,3-dimethylimidazolium iodide hemi(diiodide) (3) Refinement

**Table:**

Refinement on *F*^2^	0 restraints
Least-squares matrix: full	Hydrogen site location: inferred from neighbouring sites
*R*[*F*^2^ > 2σ(*F*^2^)] = 0.036	H-atom parameters constrained
*wR*(*F*^2^) = 0.080	*w* = 1/[σ^2^(*F*_o_^2^) + (0.0102*P*)^2^ + 8.6812*P*] where *P* = (*F*_o_^2^ + 2*F*_c_^2^)/3
*S* = 1.34	(Δ/σ)_max_ < 0.001
1912 reflections	Δρ_max_ = 0.76 e Å^−^^3^
93 parameters	Δρ_min_ = −0.97 e Å^−^^3^

### 2-Bromo-1,3-dimethylimidazolium iodide hemi(diiodide) (3) Special details

**Table:**

Geometry. All esds (except the esd in the dihedral angle between two l.s. planes) are estimated using the full covariance matrix. The cell esds are taken into account individually in the estimation of esds in distances, angles and torsion angles; correlations between esds in cell parameters are only used when they are defined by crystal symmetry. An approximate (isotropic) treatment of cell esds is used for estimating esds involving l.s. planes.
Refinement. Refinement of F^2^ against ALL reflections. The weighted R-factor wR and goodness of fit S are based on F^2^, conventional R-factors R are based on F, with F set to zero for negative F^2^. The threshold expression of F^2^ > 2σ(F^2^) is used only for calculating R-factors(gt) etc. and is not relevant to the choice of reflections for refinement. R-factors based on F^2^ are statistically about twice as large as those based on F, and R- factors based on ALL data will be even larger.

### 2-Bromo-1,3-dimethylimidazolium iodide hemi(diiodide) (3) Fractional atomic coordinates and isotropic or equivalent isotropic displacement parameters (Å^2^)

**Table:**

	*x*	*y*	*z*	*U*_iso_*/*U*_eq_	
I1	0.59338 (9)	0.17085 (4)	0.05590 (4)	0.02835 (16)	
I2	0.51595 (9)	0.40299 (5)	0.01140 (5)	0.03726 (18)	
Br	0.04393 (13)	0.28681 (6)	0.20566 (7)	0.0274 (2)	
N2	0.4459 (11)	0.3501 (5)	0.3339 (5)	0.0246 (15)	
N1	0.2322 (11)	0.4618 (5)	0.2630 (5)	0.0269 (15)	
C1	0.2538 (13)	0.3703 (6)	0.2721 (6)	0.0236 (17)	
C4	0.0471 (14)	0.5121 (6)	0.1973 (7)	0.034 (2)	
H4A	0.076771	0.518793	0.119610	0.051*	
H4B	0.032629	0.573356	0.230131	0.051*	
H4C	−0.090958	0.477458	0.198377	0.051*	
C3	0.5500 (14)	0.4327 (6)	0.3649 (7)	0.031 (2)	
H3	0.690601	0.439536	0.409197	0.037*	
C5	0.5298 (14)	0.2578 (6)	0.3667 (7)	0.0293 (19)	
H5A	0.422473	0.225817	0.406887	0.044*	
H5B	0.671533	0.263396	0.415747	0.044*	
H5C	0.551338	0.222595	0.299519	0.044*	
C2	0.4191 (15)	0.5011 (6)	0.3217 (7)	0.032 (2)	
H2	0.449093	0.565310	0.329958	0.039*	

### 2-Bromo-1,3-dimethylimidazolium iodide hemi(diiodide) (3) Atomic displacement parameters (Å^2^)

**Table:**

	*U*^11^	*U*^22^	*U*^33^	*U*^12^	*U*^13^	*U*^23^
I1	0.0248 (3)	0.0341 (3)	0.0254 (3)	−0.0032 (2)	0.0007 (2)	0.0003 (2)
I2	0.0282 (3)	0.0522 (4)	0.0320 (3)	−0.0097 (3)	0.0062 (2)	−0.0096 (3)
Br	0.0267 (4)	0.0248 (5)	0.0306 (4)	−0.0047 (3)	0.0028 (3)	−0.0036 (3)
N2	0.025 (3)	0.030 (4)	0.020 (3)	−0.005 (3)	0.005 (3)	−0.003 (3)
N1	0.030 (4)	0.024 (4)	0.026 (4)	0.001 (3)	0.002 (3)	−0.006 (3)
C1	0.031 (4)	0.021 (4)	0.021 (4)	−0.005 (4)	0.011 (3)	−0.003 (3)
C4	0.035 (5)	0.033 (5)	0.033 (5)	0.008 (4)	−0.003 (4)	0.007 (4)
C3	0.024 (4)	0.034 (5)	0.034 (5)	−0.013 (4)	0.002 (3)	−0.003 (4)
C5	0.029 (4)	0.023 (5)	0.036 (5)	0.002 (4)	0.003 (4)	0.001 (3)
C2	0.041 (5)	0.023 (5)	0.031 (5)	−0.008 (4)	0.002 (4)	−0.003 (4)

### 2-Bromo-1,3-dimethylimidazolium iodide hemi(diiodide) (3) Geometric parameters (Å, º)

**Table:**

I2—I2^i^	2.8265 (14)	C4—H4B	0.9800
Br—C1	1.858 (8)	C4—H4C	0.9800
N2—C1	1.330 (10)	C3—C2	1.331 (12)
N2—C3	1.380 (11)	C3—H3	0.9500
N2—C5	1.465 (11)	C5—H5A	0.9800
N1—C1	1.335 (11)	C5—H5B	0.9800
N1—C2	1.377 (11)	C5—H5C	0.9800
N1—C4	1.477 (10)	C2—H2	0.9500
C4—H4A	0.9800		
			
C1—N2—C3	107.3 (7)	H4B—C4—H4C	109.5
C1—N2—C5	126.7 (7)	C2—C3—N2	108.1 (7)
C3—N2—C5	125.9 (7)	C2—C3—H3	125.9
C1—N1—C2	107.6 (7)	N2—C3—H3	125.9
C1—N1—C4	126.3 (7)	N2—C5—H5A	109.5
C2—N1—C4	126.0 (7)	N2—C5—H5B	109.5
N2—C1—N1	109.4 (7)	H5A—C5—H5B	109.5
N2—C1—Br	126.8 (6)	N2—C5—H5C	109.5
N1—C1—Br	123.8 (6)	H5A—C5—H5C	109.5
N1—C4—H4A	109.5	H5B—C5—H5C	109.5
N1—C4—H4B	109.5	C3—C2—N1	107.6 (8)
H4A—C4—H4B	109.5	C3—C2—H2	126.2
N1—C4—H4C	109.5	N1—C2—H2	126.2
H4A—C4—H4C	109.5		
			
C3—N2—C1—N1	−0.1 (8)	C4—N1—C1—Br	−2.2 (11)
C5—N2—C1—N1	178.0 (7)	C1—N2—C3—C2	0.2 (9)
C3—N2—C1—Br	179.2 (6)	C5—N2—C3—C2	−177.9 (7)
C5—N2—C1—Br	−2.7 (11)	N2—C3—C2—N1	−0.2 (10)
C2—N1—C1—N2	0.0 (9)	C1—N1—C2—C3	0.2 (9)
C4—N1—C1—N2	177.1 (7)	C4—N1—C2—C3	−177.0 (8)
C2—N1—C1—Br	−179.4 (6)		

Symmetry code: (i) −*x*+1, −*y*+1, −*z*.

### 2-Bromo-1,3-dimethylimidazolium iodide hemi(diiodide) (3) Hydrogen-bond geometry (Å, º)

**Table:**

*D*—H···*A*	*D*—H	H···*A*	*D*···*A*	*D*—H···*A*
C5—H5*B*···I1^ii^	0.98	3.03	3.986 (8)	166

Symmetry code: (ii) *x*+1/2, −*y*+1/2, *z*+1/2.
